# Bee Collected Pollen and Bee Bread: Bioactive Constituents and Health Benefits

**DOI:** 10.3390/antiox8120568

**Published:** 2019-11-20

**Authors:** Rodica Mărgăoan, Mirela Stranț, Alina Varadi, Erkan Topal, Banu Yücel, Mihaiela Cornea-Cipcigan, Maria G. Campos, Dan C. Vodnar

**Affiliations:** 1 Advanced Horticultural Research Institute of Transylvania, University of Agricultural Sciences and Veterinary Medicine Cluj-Napoca, 400372 Cluj-Napoca, Romania; rodica.margaoan@usamvcluj.ro; 2 Association Health with CasaBIO, 400015 Cluj-Napoca, Romania; mirela.strant@gmail.com (M.S.); alina@casabio.ro (A.V.); 3 Apiculture Section, Aegean Agricultural Research Institute, İzmir 35661, Turkey; topalerkan@tarimorman.gov.tr; 4 Department of Animal Science, Faculty of Agriculture, Ege University, İzmir 35100, Turkey; banu.yucel@ege.edu.tr; 5 Faculty of Horticulture, University of Agricultural Sciences and Veterinary Medicine Cluj-Napoca, 400372 Cluj-Napoca, Romania; 6 Observatory of Drug-Herb Interactions, Faculty of Pharmacy, University of Coimbra, Heath Sciences Campus, Azinhaga de Santa Comba, 3000-370 Coimbra, Portugal; 7 Coimbra Chemistry Centre (CQC, FCT Unit 313) (FCTUC), University of Coimbra, Rua Larga, 3000-370 Coimbra, Portugal; 8 Department of Food Science, University of Agricultural Sciences and Veterinary Medicine Cluj-Napoca, 400372 Cluj-Napoca, Romania; dan.vodnar@usamvcluj.ro

**Keywords:** antioxidant activity, bee pollen, bee bread, cancer, diseases, health, natural product

## Abstract

Bee products were historically used as a therapheutic approach and in food consumption, while more recent data include important details that could validate them as food supplements due to their bioproperties, which support their future use as medicines. In this review data, data collected from bee pollen (BP) and bee bread (BB) essays will be discussed and detailed for their nutritional and health protective properties as functional foods. Dietary antioxidants intake derived from BP and BB have been associated with the prevention and clinical treatment of multiple diseases. The beneficial effects of BP and BB on health result from the presence of multiple polyphenols which possess anti-inflammatory properties, phytosterols and fatty acids, which play anticancerogenic roles, as well as polysaccharides, which stimulate immunological activity. From the main bioactivity studies with BP and BB, in vitro studies and animal experiments, the stimulation of apoptosis and the inhibition of cell proliferation in multiple cell lines could be one of the major therapeutic adjuvant effects to be explored in reducing tumor growth. Tables summarizing the main data available in this field and information about other bio-effects of BP and BB, which support the conclusions, are provided. Additionally, a discussion about the research gaps will be presented to help further experiments that complete the tree main World Health Organization (WHO) Directives of Efficiency, Safety and Quality Control for these products.

## 1. Introduction

Currently, there is a change in the understanding of food production and consumption and the development of functional foods is an important sector in the food market. Foods that are beyond their basic nutritional features, such as value-added or health-oriented products that positively influence our well being and quality of life are known as “functional foods”. This term underlines the positive correlation of the bioactive compounds present in these products along with health [1,2,3].

This new concept is assimilated to Hippocrates, the founder of medicine, with the nearly 2500 year old philosophy: “Let food be the medicine and medicine be the food”: paying more attention to a healthy nutrition. Nowadays, due to reasons such as an increase in treatment expenses, labor loss, an increase in life expectancy and a high percentage of populations’ aged, people desire to improve their quality of life.

Among the 21st century diseases, one of the biggest concerns is the increase of cancer. Learning to deal with the disease and providing better tools for health professionals that help patients during and after treatments will be a successful way of treatment in the future. Surgeries, chemotherapy and radiotherapy are the most used methods along with immunotherapy and molecular-targeted therapy. The multifactor associated with these protocols and with anti-cancer agents (example: drug-herb interactions, angiogenic and/or estrogen-like products, growth factors etc.) sometimes lead to uncontrolled metastatic tumors as well as high rates of adverse effects, particularly among elderly patients [4,5,6,7]. Therefore, scientists, physicians and patients with cancer make efforts and invest time to discove and develop safer and more effective future treatment schemes. The research with bee products has now come to a point that where a possible role in those protocols, namely efficacy, safety and quality control, will be assured.

Bee collected pollen (BP) and bee bread (BB) have a high nutritional value and include bioactive compounds, which have a positive effect on human health, and therefore, are regarded as “functional foods”. These products are rich in proteins, simple sugars, essential amino acids and omega fatty acids. These features strengthen immunity and help the body to fight bacteria, which will keep the body healthy, provided the body can perform a quality tissue repair [8]. Bee products are in the structure of many biochemical components found throughout functional foods, such as prebiotics, probiotics, fibre, phytochemicals (polyphenols, phenolic acids, lignans, triterpens, steroids), bioactive peptides, minerals, vitamins and organic acids. Furthermore, among all these compounds, phenolics, flavonoids and carotenoids [9] have been significantly studied in people with cancer, arteriosclerosis, weakened immune system, Parkinson’s, Alzheimer’s, cardiovascular diseases and arthritis, as well as significant preventive and therapeutic effects on the body against premature aging [10]. The main handicap of these activities is that what can be significant for some of them may be dangerous for others; for instance, the angiogenic effect is beneficial for arteriosclerosis, arthritis, Parkinson’s, Alzheimer’s and cardiovascular diseases. In cancer, they can have an anti-inflammatory effect; however, they should be carefully evaluated for the benefit-risk ratio, as this angiogenic activity can be dangerous.

In this review, firstly BP and BB composition and bioactivities are introduced, followed by in vivo and in vitro studies of the anti-cancer effects of BP and BB. Moreover, their effectiveness in the prevention and treatment of diverse anti-cancer agent-induced toxicities in animal models and patients with cancer are investigated. Additionally, we investigate the molecular mechanisms of the biological activities of BP and BB in the treatment of cancer. Finally, based on recent publications, the present use and therapeutic strategies in the near future of BP and BB are discussed.

## 2. Bee Collected Pollen (BP) Composition and Main Bioactivities

The pollen represents the male reproductive unit of flowers, and due to its high protein content, it is necessary for the nourishment of the offspring and honey bees that serve inside the hive [11,12]. The essential nutritional requirements of honey bees are similar to humans, namely proteins, carbohydrates, lipids, minerals and vitamins.

Since ancient times, BP has been recognized for its nutritional values, described by the Egyptians as “the life-giving dust”; its curative effects and its usage in human nutrition were not fully known or discovered until the development of pollen traps after the 19th century [13]. It gained attention in biochemical and medical fields when the discovery was made that it has anti-cancer and scavenging activity of reactive oxygen species (ROS), due to the presence of multiple bioactive compounds [14,15].

Historically, BP was used in the treatment of different ailments, namely as an antibiotic, in liver and kidney function or simply as a supplement of nutrients and vitamins for the human body. From all the historically known bioactivities, the therapeutic properties of specie-specific pollens are summarized in Table 1.

The above mentioned historical effects are not based on scientific or clinic studies and no connection to specific constituents has been established until now.

Currently, BP is used as nutritious food and studied for its potential therapeutic properties. In an overall screening of the published data, the extracts were studied in chronic prostatitis for their anti-androgenic bioactivity [16,17,18,19,20], such as anti-inflammatory [20,21,22], antioxidants [23,24,25] and for antimicrobial potential [23,25,26,27], as well as anti-tumor agents [28,29]. BP also shows important effects in allergies and oral desensitization [30]. The good results in immunostimulatory activity should also be considered in further research [31,32].

“Bee pollen” (BP) is a mix of bee-collected floral pollens that widely varies in composition and comprises a large number of compounds, which are cited below. All of them include proteins and free amino acids, carbohydrates, lipids including fatty acids and their esters, vitamins (as some from B-complex and E), carotenoids, folic acid and minerals; levels might differ depending on the floral origin. Flavonoids, phenolic acids and their derivatives are also important constituents, especially for their bioactivities [9,33,34,35,36,37,38,39].

The total lipid content (g/100 g BP, dry mass) of pollen is also diverse, ranging from 1 to 13 [14,40]. The high variability depends on the type of pollen and content in fatty acids, carotenoids and lipophilic vitamins [9]. The lipids present in pollen include high levels of long-chain essential fatty acids, the most abundant being linoleic, γ-linolenic and palmitic acids [9,10]. In addition, different organic acids are also found (acetic, citric, gluconic, lactic, malic, oxalic, tartaric, succinic). From these, the gluconic acid exhibits the highest concentration. For instance, Mărgăoan et al. analyzed the fatty acid composition of the total lipids in 16 BP samples from Romania. They identified 14 fatty acids, from which the most abundant were α-linolenic (32.96% on average), palmitic (25.80% on average) and linoleic (22.17% on average) acids. Based on the authors’ conclusion, the resulting percentages are based on the samples’ various botanical origins [9]. The beneficial effect of *n*-3 fatty acids in the prevention and management of cardiovascular disease, hyperinsulinemia and possibly type 2 diabetes is well known [41]. The combination of BP with high levels of α-linolenic (*n*-3) acid together with a near 1:1 ratio of *n*-6 to *n*-3 polyunsaturated fatty acids (PUFAs) proves to be a balanced source of essential PUFAs for human health. These acids exert a variety of health benefits, BP having a higher omega-3 acid value than most vegetables. As these compounds are effective in reducing platelet aggregation, they could be useful in the treatment of cardiovascular diseases. (CVD); if PUFAs are taken as a supplement and in higher doses, they should be carefully consumed by patients with cancer in order to avoid hemorrhagic episodes, especially if they will be submitted to surgical procedures [42]. Usually, doses of BP (15 g/day) do not reach the level of hemorrhagia; compared to a study which demonstrated that hemorrhagic events appear at doses of 3 g/day of omega-3 supplementation combined with warfarin or aspirin [43].

Some minor components from BP play key roles in nutrition and overall health. BP contains more than 100 enzymes and coenzymes, 16 fatty acids, all known vitamins and 3–8% mineral substances. Furthermore, flavonoids, carotenoids, over 20 trace elements, growth regulators, hormones and antioxidants are compounds that contribute to the potential bioactivities of BP in a broad-spectrum [13]. For instance, data collected from Colombian BP include levels 6.9 + 3.5 g of lipids, 23.8 ± 3.2 g proteins and total dietary fiber 14.5 ± 3.5 g. The moisture content was 7.7 ± 5.2 g/100 g and dry matter-based ash 2.5 ± 0.4 g. Fatty acids were mostly α-linolenic, palmitic and linoleic, while fructose and glucose from carbohydrates were the most concentrated main sugars. Most minerals were identified, such as K, Ca and Mg [44]. In other studies, K, Ca, Na and Mg were identified as the highest mineral contents in BP samples, as well as other metals, such as Cr, Al, Sr, Sn, Ni and V. Among trace minerals, the highest content was Mn, followed by Zn and Fe, Cu and Ni [39,45].

The relationship between the botanical origin and chemical, antioxidant and antibacterial properties is crucial for further investigations. In 2017, Velásquez et al. studied the correlation between the botanical origin, composition and antibacterial activity of multi-floral BP. In their research, *Brassica* sp. and *Galega officinalis* L. BPs showed antibacterial activity against all bacteria studied (*Escherichia coli* ATCC-25922, *Staphylococcus aureus* ATCC-25923, *Pseudomonas aeruginosa* ATCC 27853 and *Streptococcus pyogenes* I.S.P. 364-00), and the extracts surpassed the effectiveness of conventional antibiotics [23].

**Figure 1:**
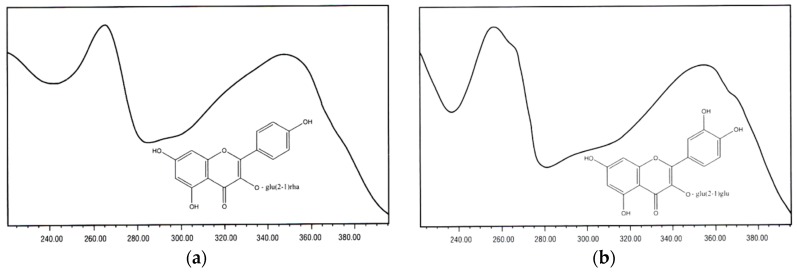
Examples of Flavonoid structures found in bee pollen (BP) and bee bread (BB), from [53] (with permission of the authors). Structure (**a**): Kaempferol-3-*O-*[rhamnosyl (1-2) glucoside] (RT (retention time) = 33.2; λ_max_ = 265, 290 sh, 320 sh, 350 nm); (**b**): Quercetin-3-*O-*[glucosyl (1-2) glucoside] (RT = 30.6; λ_max_ = 255, 266 sh, 294 sh, 355 nm).

As stated above, the antioxidant activity of BP is also related to the flora origin [46] and to its phenolic and polyphenolic compounds, such as flavonoids, among other constituents [24,47,48]. In BP samples, from diverse flora, the flavonoids tricetin, luteolin, selagin, myricetin, isorhamnetin isoquercetin, quercetin and kaempferol, were the most identified. The latter two and their glycosidic forms are the most abundant (Figure 1). They show different ratios among them, but no distinctive differences are observed in the phenolic composition [49,50,51,52,53].

The antioxidant power and scavenging activity of ROS are one of the most studied bioactivities for its broad approach. Both are significant in improving clinical research approaches of diseases such as diabetes, hypertension, obesity and cardiovascular problems, as well as in degenerative pathologies (arthritis, Alzheimer’s, Huntington’s and Parkinson’s disease) [14,15].

There is evidence that oxidative stress is the result of a concentration increase of ROS in cells that can be generated by both endogenous and exogenous factors, such as environmental factors, as well as the superoxide anion free radical O_2_●^–^. DNA and cell membrane damage is induced by increased levels of ROS; therefore, these effects are linked to cellular response and can induce chronic inflammation [54,55]. If the in vivo data corroborates with the in vitro effect of the antioxidant activity of BP substances, they may also contribute to the inhibition and removal of ROS [37], in a late sense, contributing for the reduction of the damage caused in various diseases, even in cancer.

Pollen extracts also demonstrate significant anti-inflammatory activities. In a study from 2010, BP (300 mg/kg) moderately suppressed the carrageenan-induced paw oedema. The water extract (300 mg/kg) showed minor inhibitory activity, while the ethanol extract (100 and 300 mg/kg) showed a relatively strong and significant inhibition with a mean % swelling of 48.4 and 43.5, respectively. The authors concluded that the ethanol extract shows an effective anti-inflammatory activity through the inhibition of NO production and cyclooxygenase-2 (COX-2) [56]. Additionally, BP affects the release of insulin-like growth factor I (IGF-I) and steroid hormones (estradiol and progesterone), as well as the expression of markers of apoptosis (Bcl-2, Bax and caspase-3) in rat ovarian fragments [57].

From all the cited above bioactivities below, in Table 2, the main bioactivities attributed to BP are summarized.

Based on multiple studies, the composition of Romanian BP varies in terms of macronutrients and minerals. In the study conducted by Mărgăoan et al. [73], the total polyphenols, total flavonoids and antioxidant activity were studied on six samples of fresh BP from Transylvania. Their results showed that the highest polyphenol concentration was determined in *Prunus* spp. BP (8.87 mg GAE/g), followed by *Malus domestica* Borkh. BP (7.74 mg gallic acid equivalents (GAE/g) and *Salix* spp. BP (7.69 mg GAE/g). The lowest level of total polyphenol content was obtained for BP from *Calluna vulgaris* (L.) Hull 3.76 mg GAE/g. Total flavonoids content ranged from 6.29 mg quercetin equivalents (QE)/g (*Malus domestica* Borkh.) to 2.55 mg QE/g (*Callendula officinalis* (L.) Hull).

From Greek BP samples, three different extracts (0.5 to 10 μg/mL) showed chymotrypsin-like (CT-L) proteasome activity in human fibroblasts. The water extract has been shown to exhibit important antioxidant properties and create a high CT-L proteasome activity at the concentrations of 0.5 and 2 μg/mL. The microscopical analysis of the 16 different common taxa of the Greek Flora resulted in the following species: *Papaver rhoeas* L., *Matricaria recutita* L., *Sinapis arvensis* L., *Cistus* sp., *Trifolium* sp., *Dorycnium* sp., *Cichorium* sp., *Convolvulus* sp., *Circium* sp., *Malva sylvestris* L., *Fumaria* sp., *Eucalyptus camaldulensis* Dehnh., *Anemone* sp., *Ononis* sp., *Asphodelus* sp. and *Quercus ilex* L. Greek pollen, as almost all BPs, is rich in flavonoids and phenolic acids. This composition has been reported to demonstrate the observed free radical scavenging activity on HFL-1 human fetal lung embryonic fibroblasts along with stimulation of cellular antioxidant mechanisms by other natural products. Additionally, these extracts were also tested for their antimicrobial activity against gram-positive [33].

Polysaccharides are another group of major components found in BP that are investigated as possible adjuvants for antineoplastic treatments [74,75,76].

**Figure 2:**
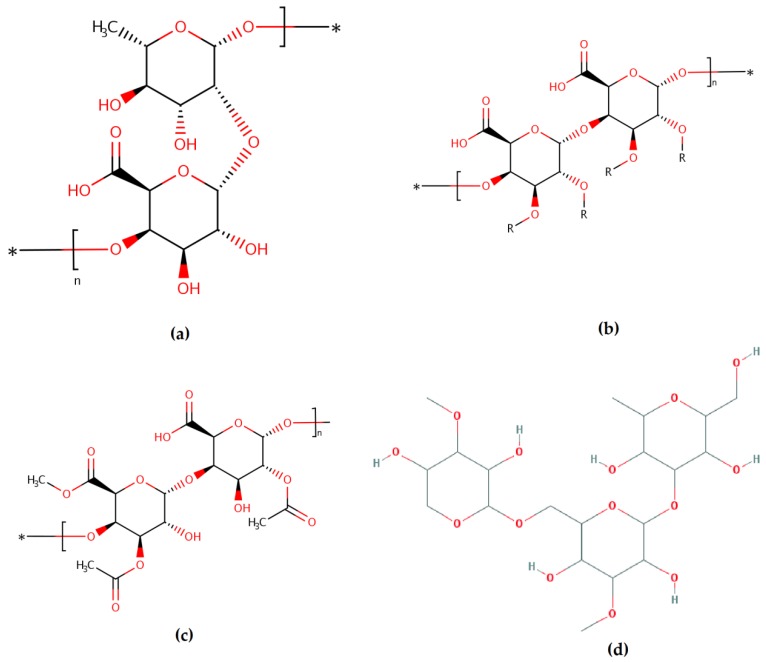
Rhamnogalacturonan type I (RG-I) (**a**) and type II (RG-II) (**b**), homogalacturonan (HG) (**c**) and arabinogalactan (AG) (**d**) structures. *—the compounds can bind to rhamnose, galactose, arabinose [29].

Previously demonstrating that BP alleviates the distress of chemotherapy-treated patients, Wang et al. investigated for the first time the antitumor activity of fractioned BP polysaccharides from *Rosa rugosa* Thunb. The acid fractions contained rhamnogalacturonan type I (RG-I) and type II (RG-II), homogalacturonan (HG) and arabinogalactan (AG) (Figure 2). All of them showed a potential in vitro antitumor activity by inhibiting the proliferation of human colon carcinoma HT-29 and HCT116 cells in a dose-dependent manner with various concentrations of BP polysaccharides for 72 h [29].

## 3. Bee Bread (BB) Composition and Main Bioactivities

“Bee Bread” (BB) is formed by adding honey and digestive enzymes to BP during its storage in the honeycomb and by fermentation of lactic acid. The titration acidity increases during the conversion of BP into BB, while the content in sytosterol and vitamins (ascorbic acid and pyridoxine) decreases. The composition of BB has a major impact when it comes to the flora in the colony’s region; it is similar to BP and varies by botanical origin. However, the identification of the main flora can also be analyzed using fingerprints of the phenolic and polyphenolic compounds performed by high performance liquid chromatography with photo-diode array detection (HPLC/DAD) assays [77].

The fatty acid content of BB is very important for honeybees, whereas PUFAs are essential for a healthy body development and productivity. Unsaturated FAs are essential for bees and for human nutrition. Therefore, this product can be a good source of all constituents mentioned above [9,78].

Data from BB composition is scarce and difficult to compare, but as an example, in the studies conducted by Nagai et al. [14], a content of about 20% protein, 3% lipids, 24–35% carbohydrates, 3% minerals and vitamins is shown. Fully balanced proteins containing all the necessary amino acids, vitamins (C, B1, B2, E, K, biotin, nicotinic and folic acid), pantothenic acid, pigments and other biologically active compounds, such as polyphenols (phenolic acid and flavonoids), carotenoids, sterols and enzymes (saccharase, amylase, phosphatases), are also present. In addition, BB contains more than 25 different micro- and macro- elements, such as Fe, Ca, P, K, Cu, Zn, Se and Mg.

Several recent studies complete the aforementioned research regarding the multitude of compounds present in BB, as following: carbohydrates (glucose, fructose, sucrose, arabinose), aliphatic acids, mainly unsaturated (α-linolenic, linoleic, oleic and 11,14,17-eicosatrienoic acids) and alkanes (C21–C35) [79,80,81,82,83,84].

The abundant polyphenols in the structure of BB are of interest from a medicinal point of view. Among polyphenols, flavonoids represent the most significant group of compounds present in BP and BB. Even though the essays carried out with BB are scarce, comparatively to BP, recently, the determination of chemical composition of ethanolic extracts (E-BB) from three different samples of BB was performed by (gas chromatography-mass spectrometry) GC/MS and the total phenolic content (33.43–36.52 mg GAE/g), antioxidant (0.56–1.11 mmol/L) and cytotoxic activities were also achieved. The effects of E-BB extracts (10, 20, 30, 50, 100 μg/mL) on the viability of the glioblastoma cell line (U87MG) were studied after 24 h, 48 h, and 72 h. A time-dependent inhibitory effect on the viability of U87MG cells was observed after 48 h incubation, with best results of EBB1 (50 µg/mL), EBB2 (100 µg/mL), EBB3 (30 and 100 µg/mL). The main inhibitory effect was observed after 72 h; in EBB1 (10 and 30 µg/mL), EBB2 (20 and 100 µg/mL) and EBB3 (30 µg/mL) [81].

Most flavonoids known as secondary components are present in the greatest amount. It has been reported that the total content of flavonoids in the ethanol extracts ranges from 10 to 166 mg/L. These compounds are predominantly found in the form of glycosides [49,85] in BP samples, except, for example, in *Eucalyptus* spp. Aglycones, where 3-*O*-methylquercetin, luteolin, tricetin and myricetin can also be identified [49,78]. These bioactive compounds are very important due to their anti-inflammatory, anti-allergic and anti-carcinogenic properties recognized by in vitro and in vivo studies. However, they also cause drug-herb interactions and association with conventional therapies should be done only when the risk is evaluated and the safety of the patient is assured [7,86].

It is well known that BB composition varies by provenance, climate conditions and seasonal variation, as well as on the melliferous species present in the respective region. Below, we describe multiple studies from different regions to demonstrate the above statement.

In data from Romania (Transylvania region) collected samples, the total phenolic and flavonoid content of BB was 7.86–3.12 mg NAE g^–1^ (Naringin Equivalent—total flavanones), 0.696–0.168 mg QE g^–1^ (Quercetin Equivalent—flavonols, flavones and isoflavones), 22.72–8.32 mg GAE g^–1^ (Gallic Acid Equivalent—total phenols) [87]. For a different group of samples from Romania (Transylvania region), the BP values for total polyphenols ranged between 20.48–10.08 mg GAE/g, 1.008–0.144 mg QE/g (flavonol, flavone and isoflavone) and for flavanone content ranged between 16.16–2.22 mg NAE g^–1^. Regarding BB samples (triplicate analyzed) the values for total polyphenols were 13.92 mg GAE/g, 0.144 mg QE/g and 12.99 mg NAE g^–1^ [88]. Cocan et al. [89] performed a study with three BP extracts and fresh BB. The content of polyphenol compounds (mg/g) in methanol extracts ranged between 25.66 (GAE) in multifloral BP and 15.33 (GAE) in BB.

In the study conducted by Baltrušaitytė et al. [90], nine BB samples, collected in Spring in Lithuania, were assessed for their antioxidant properties by the 2,2’-azino-bis-3-ethylbenzthiazoline-6-sulphonic acid (ABTS) radical cation decolourisation and 1,1-diphenyl-2-picrylhydrazyl (DPPH) radical scavenging activity. Their results showed that in the case of DPPH the values ranged between 72.5–94.0% and in the case of ABTS the values varied between 71.1–92.2%, proving to consider BB a source of natural dietary antioxidants.

Čeksterytė et al. [91] found predominant willow pollen in Spring BB samples from Lithuania (45.1 ± 3.0%), while in the Summer sample, rapeseed pollen was the main source (78.7 ± 4.5%). Twenty-two FAs were identified in these samples containing five ω-3, four ω-6 and three ω-9 PUFAs. The predominant FAs were arachidonic and oleic acids, with an average of 16.09 ± 2.38% and 15.22 ± 1.35%, respectively. The content of α-linolenic ranged mostly between 1.10% and 8.71%. The average content of the α-linolenic acid (4.32%) in all samples was significantly similar to that of docosahexaenoic acid (DHA) (4.24%). A significant difference was found in α-linolenic acid and eicosapentaenoic acid (EPA) content (7.68%). The *n*-3 DHA also present in fish oil is known to inhibit the development of non-small lung tumors through a ROS-mediated inactivation of the PI3K/Akt signaling pathway [92].

Four years later the same group [93] analyzed other BB samples, from which the rapeseed pollen varied between 54.5–80.0%, while *Salix* spp. was the secondary pollen source with 8.8–34.6%. In all samples, the highest content was found to be in α-linolenic acid (27.04–43.83%), whereas ω-6 linoleic acid content varied between 5.44% and 9.11%. Of all saturated acids, in the case of rapeseed BB, palmitic acid content was 20.5%, while arachidic acid was 2.82%. Palmitic acid (25.02–26.21%) was the highest in willow BB samples, which had 67.2–80.0% of this pollen. The highest reduction in the contents of ω-6, ω-9 and saturated FAs have been detected in wet and dry BB. The research of long-chain FAs with BB of different origin suggests that BB has more ω-3/ω-6 ratios, showing it to be more suitable for human consumption compared to other plant-derived oils.

The Colombian samples of BB presented flavonoid and phenolic content of 3.2 ± 1.0 mg (quercetin/g) and 8.9 ± 3.1 mg/g (gallic acid/g), respectively. In addition, the antioxidant activity of Ferric Reducing Ability of Plasma (FRAP) and ABTS were reported to be 46.1 ± 13.0 and 61.5 ± 10.2 mmol (trolox/g), respectively. The digestibility and bioavailability of BB were found to be significantly higher. This suggests that the nutrient effect of BB could be higher than that of BP. This potential implies a better profit of bioactive compounds for human use. According to these results, BB was mentioned by Zuluaga et al. [84] as a product that should be certified as a functional food supplement, subsequently being studied for all implementation requirements.

Ukrainian BB samples analyzed by Ivanišová et al. [83] show similar data, as presented above from other countries, with a total polyphenol content of 12.36–18.24 GA mg/g (gallic acid equivalents/dry weight) and flavonoids with the equivalent of 13.56–18.24 μg, QE-quercetin/dry weight).

Nagai et al. compared 1%, 10% and 100% solutions of hot water fraction (HWF-3 g of BB suspended and extracted by boiling with 10 volumes of distilled water), water soluble fraction (WSF-3 g of BB with 10 volumes of distilled water) and ethanol-soluble fraction (ESF-3 g of BB with 10 volumes of ethanol). The WSF under essay has shown the highest antioxidant ability. The ESF at 10% concentration was found to have the highest ability against 1,1-diphenyl-2-picrylhydrazyl (DPPH) and hydroxyl radicals [14]. Although there is a good correlation between the total polyphenol content and the resistance activity of methanol extracts, no flavonoid content correlates with any of these.

Tavdidishvili et al. [94] investigated the flavonoid compounds of BB and BP Georgian samples (Imereti region) using HPLC methods and described the naringenin, rutin and quercetin content. The quantities were determined to constitute approximately 20% of the flavonoids full content. During the storage of these products, the amount of flavonoid decreased to 6.17–5.03 g/kg.

In conclusion, a major percentage of the compounds found in BB is the provenance of BP and further research should be performed to ensure a better knowledge of the product for its efficacy and safety. Even detailed methods for quality control should be standardized.

## 4. Anti-Cancer Research with BP and BB and Its Bioactive Compounds

### 4.1. In Vitro Studies of BP and BB Correlated to the Bioactive Compounds

As described previously, the chemical composition of BP and BB may vary depending on the botanical and geographical origin, as well as the storing conditions. Polyphenols composition present in BP and BB determine their antioxidant activity, which tends to be species-specific [49,95].

Cancer is one of the main causes of mortality worldwide and a major health problem. Progress in the use of anticancer drugs is often associated with adverse reactions or recurrence. Many of these situations involve adding to the treatment of “natural products” that contribute for therapeutic failure or toxic events [95]. Therefore, therapeutic purposes can be explored with BP and BB extracts, depending of the taxa, to help eliminate these potential side effects. The variation on its chemical constituents is vast, including about 200 different substances such as free amino acids, vitamins, minerals, phenolic and polyphenolic compounds, sterols and lipids. From the latter chemical group, a special interest in unsaturated fatty acids should be granted (linoleic, linolenic and arachidonic), especially in α-linolenic acid (65.7%) found in the greatest amount in BP and BB [96]. The therapeutic effects of dietary fatty acids on cancer cell progression have been verified by in vitro and in vivo studies. PUFAs have a significant effect on the physical properties and structure of localized membrane domains. Some isolated esters of FAs have been reported to have antitumor activity against Ehrlich ascites tumor in mice [97]. Furthermore, in vivo studies should be improved to ensure the safety of future approach for this type of tumors.

Phytosterols, commonly known as plant sterols, have been shown to inhibit cholesterol absorption sites in human intestine in multiple clinical trials and also contributing to anticancer effects [98,99]. This led to researchers increased interest in phytosterols effect in human health. A fraction designated as FV-7 in the water soluble content from the pollen extract Cernilton^®^ (Cernitin SA, Lugano, Switzerland), was found to inhibit the growth of prostate cancer cell line [100]. Cernilton^®^ consists mainly of pollen extracts, L-glutamate and Stigmasterol.

Vanderplanck et al., [101] published 5 years ago the identification of sterol compounds from *Calluna vulgaris* L. Hull, *Cistus* spp., *Cytisus scoparius* (L.) Link, *Salix caprea* L. and *Sorbus aucuparia* L. pollen. The total sterols concentration for *Cistus* spp./*Cytisus scoparius* (L.) Link and *Sorbus aucuparia* L. ranged between 2.5 to 9.6 mg/g of lyophilized matter. The major phytosterols detected were β-sitosterol (SIT) and δ5-avenasterol, but significant amounts of δ7-avenasterol (in *C. vulgaris*, 20.23%) or 24-methylenecholesterol/campesterol fraction (*S. aucuparia* L., 84.07%) were also found in several pollen samples.

Nine human-derived cancer and non-cancer continuous cell lines (HEP—larynx cancer, CHANG—liver cancer, HEF—human embryo fibroblast, RT112—bladder cancer, SUZA— cancer of the testis, DU145, 1013L, LNCaP—prostate cancer, MCF-7—breast cancer) were employed to evaluate the relative in vitro activity of the pollen extract, Cernitin T-60. The results showed that the androgen-insensitive 1013L and DU145 cells demonstrated significant growth inhibition, predominantly on the 4th day. Additionally, the highest pollen concentrations (4 mg/mL) inhibited the growth of all three prostate cell lines, while rapidly depleting the cell numbers by exposure-time. The non-prostate derived cell lines showed no response to BP extract (1 mg/mL) even after the 4th day of exposure. However, the highest concentration of 4 mg/mL had a small inhibition rate on HEF and RT112 cells [102].

In 2016, Mărgăoan et al., [103] demonstrated the antiproliferative effect of *Filipendula ulmaria* (L.) Maxim BP methanolic extracts on C26 mice colorectal cancer cell lines. Their results showed that for the 6 and 12 h treatment schemes, the apoptotic index was very low (<10%). After 24 and 48 h of treatment, the index slightly increased (10–15%) for the 0.25 and 0.5 mg/mL pollen extract. The highest apoptotic index (30%) was with 1 mg/mL extract at 24 and 48 h treatment. Additionally, the apoptosis essay showed cellular shape modifications at the highest concentration (1 mg/mL) with longer extract cell exposure (24 and 48 h), which led to intra-cytoplasmatic vacuolization and granulation.

Five BB samples were screened, using in vitro assays, against different human tumor cell lines, HeLa, HepG2, MCF-7, NCI-H460, and also against non-tumor liver cells (porcine liver cells, PLP2) [104]. From all the tested samples, BB3 was the only one to inhibit the growth of all tested cell lines, solely inhibiting the growth of HepG2. BB1 and BB2 were active against MCF-7, BB4 and BB5 against NCI-H460, and principally BB4 along with BB1 and BB5 against HeLa. It should be noted that none of the BB samples showed toxicity for normal cells.

The influence of the ethanolic extracts from *Salix* spp. BB (E-BB), with and without temozolomide (TMZ) on diffuse astrocytoma cell lines (DASC), human glioblastoma multiform (U87MG) and normal human astroglia (SVGp12) was investigated. The results showed that E-BB (50 mg/mL) has stronger cytotoxic activity on U87MG cells after 72 h (26.5 of control) than TMZ alone (about 6% of control). A higher inhibitory effect on the synthesis of DNA after 24 h was found for E-BB combined with TMZ (56.4 ± 9.7%) than for the extracts alone. An inhibitory effect was observed in the cells incubated with EBB (73.6 ± 6.3%) and E-BB with TMZ (67.3 ± 5.1%) after 48 h of exposure [105]. Research by Uçar et al. [106] showed that BP shows apoptosis and affects caspase-3 activity in HL-60 cells. However, the identification of the floral origin is crucial for a better understanding of the compounds involved in the bioactivity; otherwise, it will be impossible to reproduce the anticancer effect. Among the BP and BB compounds oleanolic and ursolic acids are also identified in some species showing important antineoplastic potential bioactivities.

It is known that the vascular endothelial growth factor (VEGF) represents a key regulator of pathogenic angiogenesis in diseases such as bronchial asthma, diabetic retinopathy [107]. It is part of the structure that restores the oxygen supply to tissues when blood circulation is scarce as in hypoxic conditions [108], but when over-expressed, it can induce cancer.

Some studies aimed to investigate the citotoxicity of BP including other bee products on human umbilical vein endothelial cells (HUVECs) and cancer. In order to elucidate the mechanism of in vitro angiogenesis, VEGF-induced HUVEC proliferation and migration were examined with or without various concentrations of BP. Among the used bee products, BP showed limited effects against VEGF-induced angiogenesis, while red Chinese propolis, royal jelly and *Phoenix dactylifera* L. pollen extract had an important effect on it [109,110]. In Table 3, a summary of the main research regarding this issue is provided.

### 4.2. In Vivo Studies in Animal Models

#### Hepatoprotective Effects in Animal Experiments

Cisplatin (CP) is one of the mostly used chemotherapeutic drugs, with a wide anticancer spectrum, such as lung, prostate, testicular and ovarian cancer [113]. Indeed, CP is used as an adjuvant in radiation or post-surgery therapy [114], even though it can induce multiple side-effects, such as hepatotoxicity, nephrotoxicity, ototoxicity, neurotoxicity, nausea, vomiting and alopecia, among others [115]. The most important factors in CP-induced acute renal failure are ROS and oxidative damage. In multiple studies, CP decreased the antioxidant activity of enzymes, such as catalase (CAT), glutathione peroxidase (GPX) and superoxide dismutase (SOD). A decrease in these enzymes could lead to an increase in lipid peroxides, which leads to the formation of malondialdehyde (MDA), a decrease in antioxidant status and an increase in free radical production [116]. Additionally, CP can activate mitogen-activated protein kinase (MAPK) along with the redox-sensitive transcription factor nuclear factor kappa-B (NF-kB), which could induce inflammation, tissue injury and cell death [117,118].

Along with multiple studies that showed the antimutagenic properties of BP, in a dose-dependent manner, for certain types of cancer [119,120], a hepatoprotective effect from the extracts was demonstrated to reduce liver damage and enzymatic defects.

For future studies, it is very important to clarify whether the effect is induced by a drug-interaction. CP is metabolized by cytochrome P450 (CYP450) enzymes (mainly CYP2E1 and CYP4A11), which have an important role in drug-induced hepatotoxicity and nephrotoxicity. Overall, it has been suggested that “the cisplatin and CYP2E1 interaction leads to the generation of ROS and other oxidants resulting in renal injury; and that ROS generated by both the use of cisplatin and by the CYP2E1 increases tissue damage, induces apoptosis, and causes liver failure” [121].

The induction or inhibition of such isoenzymes by biocompounds from BP and BB as flavonoids, among others, could change the outcomes of the treatment in both sides, improve the impact of side effects and decrease the efficacy of the drug. It is important to be aware of, and do further research on, the efficiacy, which likely undergoes changes, and evaluating the amount of the available drug; the decrease implies a lower impact in the liver. Thus, this statement should be taken with care, because if a medicated patient takes a hepatoprotective product, sometimes the outcome is unpredictable, and a toxic event can happen; excepting studies regarding the administration of BP and BB in which adverse effects have not been reported [122,123].

In a study carried out with male albino mice (*Mus musculus* L.) treated with BB and Propolis extracts for 14 days, at doses of 140 and 8.4 mg/kg b.wt/day, combined with the intraperitoneal administration of CP at a dose of 2.8 mg/kg b.wt, showed a significant chemoprotective activity [119].

In order to determine the effect of BP and BB as a feed additive ingredient in mice, control group (C) and E1 were fed with 250 mg/kg pollen, while group E2 with 250 mg/kg for 21 days. The antioxidant activity was determined with a spectrophotometer, showing no adverse effects on lipid peroxidation (LPO), glutathione (GSH), superoxide dismutase (SOD), catalase (CAT), Glutathione-S-transferase (GST), Glutathione peroxidase (GP), Glutathione reductase (GR) at the tested doses of these products in mice diet. In the BP and BB administered groups, a decrease in the LPO level was observed compared to control group. The activity of GSH, SOD, CAT, GST, GR and GP in liver increased compared to control in BP and BB treated groups. Antioxidant potential of the groups treated with BB was determined to be greater [124].

A decrease in the LPO levels and antioxidant enzymes was observed in another study, BP and BB having a positive effect against bacteria when compared with antibiotics, ampicillin and amoxicillin. Thus, it has been reported that BP and BB have protective effects on *Staphylococcus aureus*-induced toxicity in the liver of mice [125].

CCI_4_ is a hepatotoxic agent that promotes the formation of free radicals that cause cellular LPO and organ damage. It has been found that *Castanea sativa* L. BP protects hepatocytes from oxidative stress and improves liver damage caused by CCI_4_ toxicity; can be safely incorporated into the daily human diet and may help reduse the risk of diseases caused by oxidative stress. It is also stated that *C. sativa* L. BP may be used as an appropriate alternative to silica in the treatment of hepatocellular pathologies [72].

The properties of BB having antimicrobial, antioxidant, prebiotic and probiotic efficacy are very important. The BB has likewise a high antioxidant and superoxide anion radical and a hydroxyl radical scavenging abilities against free radicals [126].

As it was exemplified above, these activities are common to BP and BB, and further research should be done to validate its possible use linked to certain therapies, as well as the beneficial effect of this association. In Table 4, a summary of the main research regarding this issue is provided.

### 4.3. Enzyme-Level Changes Induced by Bee Pollen

In this section, we have introduced the changes in enzyme levels for the prevention of these cancer therapy-induced toxicities (Table 5).

## 5. Potential of BP and BB in Complementary Therapies That Can Be Associated to Antineoplasic Treatments (Anxiety, Antinociceptive and Anti-Inflammatory Activities)

Anxiety is a common human mental illness. Medical treatment of this disease is associated with many side effects. Some of these drugs interfere with antineoplastic drugs, causing drug-drug interactions that will change the expected outcome for chemotherapy protocols.

For this reason, the search for new drugs with fewer side effects seems inevitable. In this study, the potential anxiolytic effects of BP hydroalcoholic extract were carried out on 20–25 g male rats in eight groups of three. Animals were injected intraperitoneally with BP hydroalcoholic solubles at doses of 200, 400, 800 and 1600 mg/kg, diazepam at a dose of 1 mg/kg and saline at a dose of 10 mL/kg. Rats receiving 800 and 1600 mg/kg hydroalcoholic BP extracts showed longer presence in the open arms of an elevated plus maze device compared to animals receiving diazepam. As a result, BP showed an anxiolytic effect of the hydroalcoholic BP extract in rats [138]. However, the identification of the botany origin of the products under essay and the compounds responsible for the bioeffects are absolutely fundamental for further investigation.

Data collected with flavonoids from medicinal plants associated to the anxiolytic effect [139,140] explains this bio-effect. The main activity is often associated to the free amino acids as glutamate, but also to the linkage of certain flavonoids, as luteolin and derivatives to the GABA_A_-benzodiazepine receptor.

The therapeutic effects of BP on glutamate excitotoxicity of the brain and glutamine-glutamate-gamma aminobutyric acid (GABA) induced by propionic acid (PPA), a short chain fatty acid to rat pups, were investigated. The results showed that the excitotoxicity measured by increasing PPA, glutamate and glutamate/glutamine ratio and lowering the ratio of GABA, glutamine and GABA/glutamate leads to multiple indications and is effective in removing the neurotoxic effects of BP to some extent [141].

Pain relief is an important part of the anticancer treatments. Nowadays, several drugs can be given, but the resistance in the effectiveness in some patients drives to a better understanding of this process and the importance of searching for new approaches.

For instance, pollen from pine (*Pinus densiflora* Siebold & Zucc.) was tested for the antinociceptive and anti-inflammatory activity in mice using carrageenan- and formalin-induced paw oedema and arachidonic acid-induced ear oedema. The ethanol extract of (100 and 200 mg/kg, per os (p.o.)) inhibited both tested phases of the formalin pain test in mice, reducing mouse writhing induced by acetic acid and elevating pain threshold in the hot plate test. The *P. densiflora* pollen extract also caused significant inhibition of carrageenan- and formalin-induced paw oedema, as well as arachidonic acid-induced ear oedema, compared with the control group. The different polyphenols found in *Pinus densiflora* Siebold & Zucc. pollen could account for the antinociceptive and anti-inflammatory actions. The results obtained indicate that the extract possesses analgesic and anti-inflammatory effects [66].

Even though it is known that *Pinus* pollen is not usually part of the floral sources associated with BP or BB, the data investigated until now give important information about its anti-inflamatory and antioxidant acitivities and significant flavonoids content. Given that these compounds exist in virtually all of the samples studied to date of BP and BB, their potential appearing to be immense, and should be largely explored in the future.

The result of one study showed that BB supplementation diet of rabbits improved wound healing parameters, such as wound tensile strength, neovascularization and fibroblast count in the incision wound, but with no significant difference in the epithelization and hydroxyproline content of the supplemented group compared to the control. This experiment indicated the possibility of using BB in malnourished patients to improve surgical outcomes [142]. However, as highlighted above, this bioactivity is mainly related to the angiogenic process and could be dangerous for the patient. Again, the evaluation of the benefit/risk should be carefully performed.

Inflammation and oxidative stress are closely related with anti-cancer agent-induced toxicities [72,132,137]. Regarding inflammation, it has been reported that BP lowered the levels of interleukin-6 (IL-6) and tumor necrosis factor-alpha (TNF-α) compared to β-estradiol-fed rats [137].

It has been established that pollen grains contain NAD(P)H oxidases that induce oxidative stress in the airways, thus leading to the development of allergic inflammation.

GSH, glutathione peroxidase (GSH-Px), glutathione-S-transferase (GST) and SOD are well-known endogenous anti-oxidants and anti-oxidant parameters. Increased activities and levels of these factors were detected in the liver, kidney and testis of rats and mice treated with CP and BP compared to only CP-treated ones [132,133]. Moreover, increased levels of SOD were detected in the liver and plasma of rats treated with BP compared to those administered CCl_4_ alone [72,131,135]. Additionally, these studies also showed that levels of malondialdehyde (MDA), commonly used as an oxidative stress biomarker, were significantly lowered in the kidney, liver, heart and brain tissues of rats and mice treated with propoxour or CP and BP, compared to those administered CP alone [71,132,133]. Decreased levels of this factor were detected in the liver of rats and mice treated with BP compared to those treated with CCl_4_ alone [72,131,135].

In rats and mice treated with BP, the MDA levels in the liver and kidney were significantly lowered compared to the groups treated with CCl_4_ or CP alone [72,131,132,133,135].

The GSH-Px levels in the liver, kidney, heart and brain were significantly higher in rats administered with propoxour and BP than those administered propoxour only [71]. Similar findings were also reported in the liver of mice treated with CCl_4_ [131].

As referred above CP can induce hepato- and nephro-toxicity in patients that undergo this treatment. Huang et al. [133] found that the GSH levels in the kidney and liver of rats treated with BP and CP were significantly higher compared to those treated with CP alone. Similar findings were reported in the prostate and testis of rats treated with BP.

BB in aluminium toxicity: aluminium, blood supply, transaminase, C-reactive protein and monocytes have caused a significant rise in the number of levels, and significantly decreased haemoglobin. These changes have improved significantly by BB administration. It has antioxidant features and has demonstrated a protective effect on the elevation of C-reactive protein, leukocytes and monocytes of blood and hepato-renal toxicity and inflammatory markers of aluminum-borne [143].

## 6. Clinical Trials

The positive effects of functional food can either maintain a welfare and health condition or reduce the risk of pathological consequences. Adverse and unwanted side effects of foods are important for human health, especially unprocessed functional foods. With the increase in environmental humidity and temperature, the total number of coliform can increase due to untreated BP consumption. This aspect may be challenging for human and animal health, due to many microorganisms that may be harmful to body health [144]. On the other hand, BB consumption proves to be more efficient, due to lactic fermentation that enhances storage longevity and increases digestibility.

In the samples of BP and BB collected from Transylvania, it was found out that K tested from the BP and BB samples has the highest concentrations, followed by Ca and Mg. Noteworthy oligo-elements, such as Fe and Zn, were also found. These results confirm that Romania’s BP and BB can be used by people as a natural mineral source [145].

Thus, BP and BB may be excellent candidates for future studies involving BP flavonoids, phytotherapy, molecular pharmacology, allergic and immunotherapeutic chemicals and antibacterial and antitumor potential. Additionally, many studies suggest that phenolic compounds are the main active agents of these products, but not the only ones [32,146].

### Chronic Prostatitis

Chronic prostatitis (CP) is one of the most frequent disease in men aged over 50, with different clinical presentations [147,148]. Class III chronic prostatitis/chronic pelvic pain syndrome (CP/CPPS) is the most frequent category in accordance to the National Institute of Health (NIH) classification [149]. Additionally, available therapies and therapeutic efficacies are scarce and require further in-depth analysis, also due to recent discovery that CP might defect semen quality. Therefore, a noteworthy investigation needs to be done in pathogenesis and alternate medication of CP.

Oxygen free radicals (OFR), which cause tissue damage by LPO [150], comprise mainly superoxide free radical anion (O_2_•¯), hydrogen peroxide (H_2_O_2_), nitrogen monoxide (NO) and hydroxy free radical (•OH). Destruction of cellular segment is a result of LPO yield of multiple types of secondary free radicals and reactive compounds. With all these, cells are equipped with antioxidants, such as vitamin C and E, CAT, GSH, SOD, which can act as defensive mechanisms against the cytotoxicity and supernumerary of OFR [151]. Thus, OFR play an important role in pathogenesis of CP and infertility.

BP of *Brassica rapa* L. is widely used as a natural food supplement in China and as a herbal medicine that strengthens the body’s resistance to diseases, including cancer. A steroid fraction of the chloroform extract from BP of *B. rapa* L. could trigger cytotoxicity by inducing apoptosis. *B. rapa* L. shows that the steroid fraction of chloroform extract from BP may be a promising candidate for the treatment of advanced prostate cancer [64].

Promising results of pollen extracts in the treatment of chronic prostatitis, prostatodynia (prostate pain) and benign prostatic hypertrophy (BPH) have been obtained. Pollen Extract Cernilton^®^ has been used in chronic prostate treatment for many years and has positive results. The fatty part of the pollen extract inhibits prostaglandin synthesis by inhibiting the enzyme lipoxygenase and cyclooxygenase in the eicosanoid chain. This results in an anti-inflammatory effect. Pollen extracts have a selective and specific effect on prostate. Experimentally, pollen extracts inhibit the growth of prostate cells in cell cultures. It has been established that this inhibition is related to the dose and duration of treatment.

For treatment of chronic prostatitis syndrome, 26 (36%) patients were treated with a pollen extract Cernilton^®^ at a dose of 1 tablet tid for 6 months, 30 (42%) had a flow rate increase, leucocytosisin post-prostatic massage urine and ejaculate in C3/koeruloplasmin complex. Cernilton^®^ has been reported to be well tolerated in 97% of patients [16]. Another study evaluated the effect of pollen extract on the lower urinary tract symptoms of the preparation Prostate/Poltit in patients with chronic nonbacterial prostate/chronic pelvic pain syndrome. In the general clinical evaluation of the treatment outcome, pollen extract preparation after treatment was reported to be more effective in patients treated with prostate/poltit than placebo-treated patients [19,20].

Additionally, the efficacy of Cernilton^®^, a rye-grass pollen extract versus placebo in men with category IIIA CP/CPPS was evaluated in a study consisting of 93 patients divided into two groups (Cernilton^®^ to Cernilton^®^, *n* = 48; placebo to Cernilton^®^, *n* = 45) up to 24 weeks. The results clearly showed that the pain, quality of life domains and the total NIH-CPSI score improved significantly at week 12 in the Cernilton^®^ group versus placebo and continued to improve at week 24 in both groups [20].

Qian et al. [152] followed the research with the evaluation of the therapeutic efficacy of Cernilton^®^ in BPH patients with histological prostatitis after transurethral resection of the prostate (TURP). They concluded that Cernilton terapy may improve lower urinary tract symptoms in patients with moderate prostatitis, as well as sexual dysfunction in patients with severe prostatitis.

Night sweats, pain, hair loss, forgetfulness, depression and sleep disorders are common problems in breast cancer patients who are subjected to anti-hormonal treatment. Evidence has been provided that honey and BP mixture can improve the menopausal symptoms of breast cancer patients receiving anti-hormonal therapy [153].

In Table 6 clinical studies regarding the effect of BP and BB administration in patients with chronic prostatitis or chronic pelvic pain syndrome are reported.

In the European Association of Urology guidelines, phytotherapy, including Cernitin pollen extract (Cernilton^®^), is recommended for patients with inflammatory prostate pain syndrome, with no known reports of severe adverse events associated with its administration. If such a non-antimicrobial drug can effectively reduce serum prostate specific antigen (PSA) in prostate biopsy candidates with chronic inflammation of the prostate, an optimal method including that could be developed for avoidance of an unnecessary biopsy“ [160]. Thus, to verify this possibility, Togo et al. [160] administered Cernitin pollen extract tablets to prostate biopsy candidates for 30 days, before carrying out a prostate biopsy procedure. Then, they evaluated the relationship between the reduction in serum PSA levels and prostate biopsy outcomes. The authors suggested that effective protocols using Cernitin pollen extract have potential in avoiding unnecessary prostate biopsy procedures in patients with elevated prostate-specific antigen. Further studies are required to confirm their findings in order to develop an extensive protocol that can be used on a large number of participants.

## 7. Therapeutic Strategies for BP and BB and Future Perspectives

In today’s world, as Api-Nutrition combined with natural and healthy foods is gaining importance, more research and innovation is required on the production, consumption and health effects of bee products, particularly in the therapeutic field.

Compared to other natural products, BP and BB have the advantage to bring a significant amount of nutrients that meet the human body needs, which is a premise for optimal functioning, a good functioning of the immune system and resistance against illnesses, as well as supporting the healing processes in the body.

BP and BB have been studied for their promising potential agents in cancer therapy. As mentioned above, their inhibitory effect, such as the prevention of tumor growth, has been confirmed by in vitro and in vivo studies, in animal experiments and in certain types of cancer, such as prostate cancer, but with standardized extracts. With all these, many clinicians and researchers claim that the anti-cancer effect exerted by BP are not satisfactory for the improvement in terms of prognosis and survival rates, especially when it comes to the scarce publications regarding BB. In fact, a controlled clinical essay should be performed in order to ensure the Efficacy versus the Safety, regarding other potential extracts from more active floral sources in this area.

Other investigations showed that various fractions of BP are potential therapeutic agents for different types of cancers, for example, a steroid fraction of chloroform extract from *Brassica rapa* L. BP (CPBC) was tested on human cancer cell viability. Protein expression was detected by rabbit polyclonal anti Bcl-2 antibody and secondary antibody (goat anti-rabbit immunoglobulin G (IgG)) conjugated with peroxidase. The study showed that among all nine cancer cell lines of different origin, the steroid fraction displayed the strongest cytotoxicity in human prostate cancer PC-3 cells. After the treatment, an obviously enhanced Caspase-3 activity was observed, along with a time-dependent decrease in the expression of anti-apoptotic protein Bcl-2 [64]. In addition, this study also showed that the cells derived from human prostate (PC-3, LNCaP) proved to be more sensitive to the treatment than those of non-prostate origin, suggesting that CPBC may be a selective cytotoxic agent of human prostate-derived cancer cell lines.

Plant sterols have been proven to exert anticancer effects in multiple cell lines, which inhibited the growth of MDA-MB-231 and HT-29 cells cells [101]. Currently, brassinolide is gaining ground in the medical field. It is a naturally occurring plant hormone that promotes growth, increases yields for grain and fruit crops, and makes plants resistant to drought and cold conditions. Brassinolide, from *Brassica napus* L. pollen, was isolated to investigate the cell viability effect on androgen-independent human prostate cancer PC-3 cells. The cells were treated with different concentrations of brassinolide (0, 10, 20 and 40 mM) for 12, 24 and 36 h. The results clearly showed that a 12 h interval induced a concentration dependent increase in the apoptotic rate and an increase in caspase-3 activity [64]. As human prostate cancer was shown to be highly carcinogenic and metastatic, hormone unresponsive and resistant to normal rates of apoptosis, brassinolide proves to be an efficient approach for future studies regarding its effectiveness in prostate cancer and other diseases. Recently, the apoptotic effect of this plant sterol was tested on drug-resistant (VPA17) and drug-sensitive (H69) SCLC (small-cell lung carcinoma) cells, with high cytotoxic effect (IC_50_ = 2 μM) after 24 h, proving to be pharmacologically active in both drug-sensitive and drug-resistant SCLC cells [161].

Previously, in this paper, we introduced combined therapies of BP and other anti-cancer agents in the treatment of prostate cancer. For example, temozolomide (TMZ), an oral alkylating agent that belongs to imidazotertrazines [162], is known to exhibit anti-cancer properties [163,164], recently tested in human glioma cells. The combination of TMZ and BB exerted strong cytotoxic activity on human glioblastoma multiforme cells (U87MG) than TMZ alone.

Additionally, in a recent study, the activity of Graminex pollen (assortment of standardized pollen of *Secale cereal* L., *Zea mays* L. and *Phleum pretense* L.) on prostate cells (PC3) and in rat prostate specimens along with *E. coli* lipopolysaccharide (LPS) was evaluated. A significant cytotoxic effect on prostate PC3 cancer cell lines was observed at the highest tested concentration (500 μg/mL), Graminex pollen reducing ROS production by PC3 cells and MDA, NF-κB mRNA and prostaglandin E_2_ (PGE_2_) levels in rat prostate specimens [165]. Other examples should be explored to amplify the possibilities in cancer treatments.

## 8. Conclusions

In this review, summarized studies on the main bioactivities of BP and BB, correlated to cancer research, were reported based on in vivo and in vitro studies.

Countless reports demonstrated that BP and BB could be explored to protect against anti-cancer agent-induced toxicities, particularly in liver and kidney fibrosis. Furthermore, specific extracts can do modulation of various biological activities involving protection via the anti-oxidative activity and inflammation, which is closely associated to some constituents, for example, phenolic and polyphenolic compounds found in BP and BB.

Therefore, a strong hypothesis for the safety incorporation of these products into the daily human diet as a food supplement is needed. For instance, *Castanea* and *Brassica* BP would increase the inhibition of inflammation and oxidative stress, respectively. Several clinical studies have confirmed the efficacy of BP and BB against drug-induced toxicities; however, further research should be carried out, as most of them show studies with small population groups, either in animal or in patients. Therefore, more detailed examinations are vital for discussing the clinical efficacy of BB and BP in these patients, with long term treatment, and research regarding chronic toxicity. Data for drug-herb (pollen/extracts) are still scarce and will prove to be significantly important in future treatment protocols. Although multiple problems remain unsolved, we consider that these bee products are useful tools for the prognosis and improvement of patients’ quality of life with various diseases and even in anti-cancer therapies/therapeutic protocols that should be validated for their safety.

## Figures and Tables

**Table 1:** Therapeutic properties of different pollen types in folk medicine (after [13]).

Properties	Bee Pollen Type
Antibiotic	*Castanea* spp., *Eucalyptus* spp., *Taraxacum* spp., *Trifolium* spp., *Zea mays* L.
Anti-atherogenic	*Aesculus hippocastanum* L., *Castanea sativa* Mill., *Prunus* spp., *Salix* spp.
Anti-anemia	*Acacia* spp., *Citrus* spp., *Crataegus* spp., *Papaver* spp., *Tilia* spp.
Antitussives	*Papaver* spp.
Diuretic	*Centaurea cyanus* L., *Prunus* spp., *Taraxacum* spp.
Digestive	*Acacia* spp., *Lavandula* spp., *Rosmarinus officinalis* L.
Cardioprotective	*Crataegus* spp.
Hepatoprotective	*Aesculus hippocastanum* L., *Castanea sativa* Mill., *Cystus incanus* L., *Prosopis juliflora* (Sw.) DC., *Schisandra chinensis* (Turcz.) Baill., *Taraxacum* spp.
Kidney function	*Brassica napus* L., *Phoenix dactylifera* L., *Schisandra chinensis* Turcz.) Baill., *Trifolium alexandrinum* L., *Zea mays* L.
Immunomodulating	*Eucalyptus* spp., *Malus* spp.
Ulcer healing	*Brassica napus* L.

**Table 2:** Current therapeutic properties of different bee pollen and bee bread type.

Functional Properties	BP and BB Type	Extract Type or Concentration	Bioactivity	References
Anti microbial	*Castanea sativa* Mill.	10 g of *Castanea sativa* Mill pollen (A1–A5, E1–E4) extracted by 100 mL of methanol from nine different populations	Inhibition zone diameter: A1 (9–21 mm) against *Candida albicans* ATCC 14053, *Bacillus cereus* 7064, *Enterococcus faecalis* ATCC 51299, Methicillin Resistant *Staphylococcus aureus* (MRSA), *Micrococcus luteus, Staphylococcus aureus* ATCC 6538; A2 (9–18 mm) against *Candida krusei* ATCC 6258, *Escherichia coli* ATCC 11293, MRSA, *M. luteus, S. aureus*; A3 (9–20 mm) against *C. krusei, E. coli,* MRSA, *M. luteus, S. aureus*; A4 (9–21 mm) against *C. parapsilosis, B. cereus, E. faecalis,* MRSA, *M. luteus, S. aureus*; A5 (9–21 mm) against *E. coli,* MRSA, *M. luteus, S. aureus*; E1 (9–23 mm) against *C. krusei, Candida parapsilosis* ATCC 22019, *B. cereus*, MRSA, *M. luteus, S. aureus,* ancomycin Resistant *Enterococcus* (VRE); E2 (9–22 mm) against *C. krusei, C. albicans*, *B. cereus*, *E. coli*, MRSA, *M. luteus, S. aureus;* E3 (12–21 mm) against *C. krusei*, MRSA, *M. luteus, S. aureus*; E4 (10–21 mm) *C. krusei, C. albicans*, *C. parapsilosis*, *B. cereus*, MRSA, *M. luteus, S. aureus*	[58]
	*Ranunculus sardous* Crantz., *Ulex europaeus* L.	N/S	Marked antibiotic activity against *Pseudomonas aeruginosa* due to herbacetin derivates	[59]
	*Brassica napus* subsp. *napus* L.	10 g of pollen extracted in 99.9% and 70% (*v/v*) methanol (MEh and MEl) and 96% and 70% (*v/v*) ethanol (Eh and El)	MEh and MEl: 2.33–3.00 mm against *Listeria monocytogenes* CCM 4699; 1.33–2.66 mm against *Pseudomonas aeruginosa* CCM 1960; 2.33–3.67 mm against *Staphylococcus aureus* CCM 3953; 2.00–3.83 mm against *Salmonella enterica* CCM 4420; 1.67–3.00 mm against *Escherichia coli* CCM 3988; Eh and El: 2.33–3.67 mm against *L. monocytogenes*; 1.67–3.67 mm against *P. aeruginosa*; 2.33–3.00 mm against *S. aureus*; 2.00–3.00 mm against *S. enterica*; 2.33–3.67 mm against *E. coli;*	[60]
	*Helianthus annuus* L.	10 g of pollen extracted in 99.9% and 70% (*v/v*) methanol (MEh and MEl) and 96% and 70% (*v/v*) ethanol (Eh and El)	MEh and MEl: 2.33–3.67 mm against *L. monocytogenes*; 2.00–2.67 mm against *P. aeruginosa*; 1.67–2.00 mm against *S. aureus*; 2.67–3.33 mm against *S. enterica*; 1.33–2.67 mm against *E. coli*; Eh and El: 2.33–2.67 mm against *L. monocytogenes*; 1.00–2.67 mm against *P. aeruginosa*; 1.00–2.67 mm against *S. aureus*; 2.17–3.67 mm against *S. enterica*; 1.67–2.67 mm against *E. coli;*	[60]
	*Papaver somniferum* L.	10 g of pollen extracted in 99.9% and 70% (*v/v*) methanol (MEh and MEl) and 96% and 70% (*v/v*) ethanol (Eh and El)	MEh and MEl: 0.67–2.67 mm against *L. monocytogenes*; 1.00–1.67 mm against *P. aeruginosa*; 1.67–2.50 mm against *S. aureus*; 1.67–2.67 mm against *S. enterica*; 2.00–2.67 mm against *E. coli*; Eh and El: 2.00–2.33 mm against *L. monocytogenes*; 1.00–1.67 mm against *P. aeruginosa*; 1.67–3.67 mm against *S. aureus*; 1.67–2.17 mm against *S. enterica*; 1.83–3.00 mm against *E. coli;*	[60]
	BB—predominant *Bupleurum spinosum* Gouan.; *Anethum graveolens* L.	Hydro methanolic BB extract of 20 mg/mL in water	MIC: 0.04 mg/mL against *B. cereus*; 0.25 mg/mL against *E. coli*; 0.175 mg/mL against *S. aureus, L. monicytogenes, Enterobacter cloacae, Salmonella typhimurium*; MBC: 0.08 mg/mL against *B. cereus*; 0.35 mg/mL against *S. aureus, L. monicytogenes, E. coli, E. cloacae, S. typhimurium*	[61]
	BB—predominant *Bupleurum spinosum* Gouan.; *Anethum graveolens* L.	Hydro methanolic BB extract of 20 mg/mL in water	MIC: 0.35 mg/mL against *Aspergillus* *ochraceus*; 0.50 mg/mL against *Aspergillus* *fumigatus*, 0.70 mg/mL against *Penicillium* *funiculosum*; 1.00 mg/mL against *Aspergillus* *niger*, *Penicillium ochrochloron, Penicillium verrucosum var. cyclopium*; MBC: 0.70 mg/mL against *A. ochraceus*; 1.00 mg/mL against *A. fumigates, P. funiculosum;* 1.40 mg/mL against *A. niger*, *P. ochrochloron, P.v. cyclopium*	[61]
Antioxidant	Selected monofloral species	2 g of BP extracted in 15 mL methanol	DPPH value ranging between: 0.135–2.814 mmol Trolox g^–1^, in *Pinus* spp. and *Salix* spp.; TEAC value ranging between: 0.546–6.838 mmol Trolox g^–1^, in *Pinus* spp. and *Salix* spp. 0.255–5.355 mmol Fe(II) g^–1^, in *Knautia arvensis* (L.) Coulter and *Matricaria chamomilla* L.	[47]
	BB—predominant *Bupleurum spinosum* Gouan.; *Anethum graveolens* L.	1 g of BB stirred with 30 mL methanol/water (80:20 *v/v*) mixture and prepared at a final concentration of 20 mg/mL in water	Total antioxidant capacity (mg AA/g extract) 143 ± 22 DPPH assay (EC_50_, mg/mL) 0.98 ± 0.06 ABTS assay (EC_50_, mg/mL) 0.50 ± 0.04 Reducing power (EC_50_, mg/mL) 0.19 ± 0.03	[61]
	Selected monofloral species	0.25 mL BP in 80% methanol	Total antioxidant activity (%): 6.8–86.4 in *Zea mays* L. and *Sinapis alba* L. DPPH value (%): 8.6–91.3 in *Lamium purpureum* L. and *Aesculus hippocastanum* L. HRSA (%): 10.5–98.0 in *Aesculus hippocastanum* L. and *Pyrus communis* L.	[62]
	*Helianthus annus* L.	0.5 g of pollen extracted with 10 mL of 80% methanol and 50% ethanol	Methanolic extract: TPC: 816 mg/kg GAE of DW; TFC: 843 mg/kg QE DW; ABTS radical scavenging activity: 95.5%; Ethanolic extract: TPC: 2907 mg/kg GAE of DW; TFC: 865 mg/kg QE DW; ABTS radical scavenging activity: 75%	[63]
Anti-carcinogenic	*Brassica rapa* L.	1.95 g pollen fraction (chloroform extract) with 12.5, 25, 50 and 100 μg/mL of pollen extract administered for 24 h	Citotoxicity in MCF-7, Hela, BEL-7402, BCG-823, KB, A549 and HO8910 cells with 100 μg/mL extract ↑ caspase-3 enzyme activity; ↓ expression of anti-apoptic proteins Bcl-2	[64]
	*Cistus x incanus* L., *Salix alba* L.	1 g bee pollen mixed with 9 mL 70% ethanol with final concentrations of: 1 mg/mL, 10 mg/mL and 100 mg/mL at 24 h until harvest (72 h)	*C. incanus* extract 2 induced toxicity at 355.6 mg/mL *S. alba* extract 2 induced toxicity at 660 mg/mL, (91.82–7.46%, Inhibition of 17-β estradiol activity	[65]
Anti-inflammatory	*Pinus densiflora* Siebold & Zucc.	Three times extracted pollen with 70% ethanol, with orally administered dose of 100 and 200 mg/kg	Significant effect in formalin test of mice with pollen (100 and 200 mg/kg) at the first (0–5 min) and second phase (15–30 min); Dose of 200 mg/kg delayed the response of mice to hot plate thermal stimulation; ↓ inflammation induced by carrageenan, formalin and arachidonic acid, due to flavonoid content in *Pinus* pollen	[66]
	*Cistus spp*.	200 g of BP extracted with water or 95% ethanol; with orally administered dose of BP (300 mg/kg), Water BP (300 mg/kg), EtOH BP (100 and 300 mg/kg)	↓ inhibition of carrageenan-induced edema at 300 mg/kg water PB; ↑ inhibition of carrageenan-induced edema at 100 and 300 mg/kg (48.4% and 43.5%) ↑ anti-inflammatory effect of ethanol extract, strong inhibition of carrageenan-induced paw edema ↑ inhibition of COX-1 and COX-2 in water BP (IC_50_: 150 μg/mL and 10.3 μg/mL) ↑ inhibitionof COX-1 and COX-2 in ethanol BP (IC_50_: > 150 μg/mL)	[56]
Anti-osteoporosis	*Cistuscreticus* L.	5 g of BP in 20 mL distilled water, with concentrations of 10, 100, 1000 µg/mL	↑ calcium content (mg/g dry bone) on VD_3_-induced decrease, in the femoral-diaphyseal and metaphyseal tissues by BP dose increase ↑ calcium content (mg/g dry bone) on PGE_2_-induced decrease, in the femoral-diaphyseal and metaphyseal tissues by BP dose increase ↓ calcium content (mg/g dry bone) on PTH-induced decrease, in the femoral-diaphyseal and metaphyseal tissues by BP dose increase ↓ glucose consumed (mg/g dry bone) on PTH-stimulated glucose consumption in the femoral-diaphyseal and metaphyseal tissues by BP dose increase ↓ lactic acid production (mg/g dry bone) on PTH-stimulated lactic acid production in the femoral-diaphyseal and ↑ lactic acid production (mg/g dry bone) in the metaphyseal tissues by BP dose increase ↓ TRACP (nmol/min/mg protein) on PTH-induced increase in TRACP activity in the femoral-diaphyseal and metaphyseal tissues by BP dose increase	[67]
	*Cistuscreticus* L.	5 g of BP in 20 mL distilled water, with concentrations of 10, 50 and 100 µg/mL BP extracts fractioned to less than MW 1000 (A), from MW 1000 to MW 10,000 (B) and greater than MW 10,000 (C)	↑ calcium content (mg/g dry bone) in rat femoral-diaphyseal tissues in the presence of 50 μg/mL BP extract (< MW 1000) and moderately higher in the presence of 100 μg/mL in all BP fractioned extracts ↑ calcium content (mg/g dry bone) in rat femoral-diaphyseal and -methaphyseal tissues by dose increase of 25 and 50 μg/mL BP extract (< MW 1000) ↑ osteoclast-like MNCs (number/culture) on PTH-induced osteoclastic cell formation by dose decrease (10 and 50 µg/mL) and higer fractioned extracts	[68]
	*Cistuscreticus* L.	5 g of BP in 20 mL distilled water (oral administration) 20 g of BP in 99.5% ethanol (30 mL) for use on tissues of rats; Concentrations: 1, 5 or 10 mg/mL 100 g body weight orally administered to rats for 7 days; 10, 100 and 1000 µg/mL water and ethanol extracts	↑ calcium content (mg/g dry bone) in the femoral-diaphyseal and metaphyseal tissues by oral administration of BP water extracts (5 and 10 mg/mL/100 g body weight) ↑ calcium content (mg/g dry bone) in the femoral-diaphyseal and metaphyseal tissues in the presence of water-solubilized extract (100 or 1000 µg/mL) and ethanol extract (1000 µg/mL) by dose increase ↑ calcium content (mg/g dry bone) in the femoral-diaphyseal tissues with water-solubilized extract (100 µg/mL) ↑ alkaline phosphatase (µmol/min/mg protein) activity and DNA (mg/g wet bone) content in the presence of water-solubilized extract (100 or 1000 µg/mL)	[69]
	*Brassica napus* L., *Camellia sinensis* (L.) Kuntze., *Fagopyrum esculentum* Moench.	5 g of BP in 20 mL distilled water (oral administration) 20 g of BP in 99.5% ethanol (30 mL) for use on tissues of rats; Concentrations: 1, 5 or 10 mg/mL 100 g body weight orally administered to rats for 7 days; 10, 100 and 1000 µg/mL water and ethanol extracts	↑ calcium content (mg/g dry bone) in the femoral-diaphyseal or metaphyseal tissues in the presence of water-solubilized extract (100 µg/mL), best results in *C. sinensis* (L.) Kuntze (> 240 mg/g dry bone calcium content)	[69]
	*Cistuscreticus* L.	5 g of BP in 20 mL distilled water, with final concentrations of 5, 10 or 20 mg/mL 100 g body weight orally administered to rats for 14 days	↑ calcium content in the femoral-diaphyseal (5, 10 mg/100 g) or metaphyseal (5, 10 or 20 mg/100 g) tissues in the presence of water-solubilized extract (5, 10 or 20 mg/100 g) in STZ-diabetic rats ↓ serum glucose (mg/dL) concentration by BP dose increase ↓ triglyceride concentration ↑ alkaline phosphatase (µmol/min/mg protein) activity and DNA (mg/g wet bone) content in the presence of water-solubilized extract (5, 10 or 20 mg/100 g) by dose increase ↓ serum calcium (mg/dL) concentration in STZ-diabetic rats by BP dose increase ↑ inorganic phosphorus (mg/dL) concentration in STZ-diabetic rats by BP dose increase	[70]
Hepatoprotective	*Brasssica napus* L.	30 g of BP in 100 mL distilled water, with orally administered concentrations of: G1 (control): 1mL of distillated water + 1 mL SO; G2: 100 mg/kg/bw/day of WSBP +1 mL SO; G3: 20 mg/kg/bw/day propoxur in 1 mL distilled water and 1 mL SO; G4: 20 mg/kg/bw/day propoxur in 1 mL volume of soy oil and with 100mg/kg/bw/day of WSBP orally administered for 14 days	↓ plasma and tissue (liver, kidney, brain, heart) MDA (nmol/mL-nmol/mg-prot) levels in the WSBP-treated group (G2) and propoxur + WSBP (G4) compared to propoxour treated ones (G3); ↑ erythrocyte and tissue (liver, kidney, brain, heart) SOD (U/mg Hb-U/mg-prot) activities in WSBP-treated group (G2) and Propoxur + WSBP (G4) compared to propoxour treated ones (G3); ↑ erythrocyte and tissue (liver, kidney, brain, heart) GSH-Px (U/mgHb-U/mg-prot) activities in WSBP-treated group (G2) and propoxur + WSBP (G4) compared to propoxour treated ones (G3); ↑ serum T-protein and albumin (mg/dL) levels in WSBP-treated group (G2) and propoxur + WSBP (G4) compared to propoxour treated ones (G3); ↓ creatin (mg/dL) levels WSBP-treated group (G2) and propoxur + WSBP (G4) compared to propoxour treated ones (G3)	[71]
	*Castanea sativa* L.	1 g of BP with 10 mL methanol, with orally administered concentrations: G1 (control): 0.9% NaCl (i.p.); G2 (control): 0.8 mL/kg OO (i.p.); G3 (control) 0.8 mL/kg Ethanol (i.p.); G4 (CCI_4_) 0.8 mL/kg CCI_4_ in OO; G5 (Silibinin) 0.8 mL/kg CCI_4_ in OO + Silibinin (50 mg/kg/day) gavage; G6 (low BP) 0,8 mL/kg CCI_4_ in OO (i.p.) + BP (200 mg/kg/day) gavage; G7 (high BP) 0,8 mL/kg CCI_4_ in OO (i.p.) + BP (400 mg/kg/day) gavage	Protective effect of hepatocytes from oxidative stress Healing of liver damage induced by CCI_4_ (carbon tetrachloride) toxicity ↓ weight loss % in G6 (low pollen) and G7 (high pollen) compared to G4 (CCI_4_) G5 (silibinin) and G1-3; ↓ plasma AST, ALT (U/L) and MDA (nmol/mL plasma) levels in G5 (silibinin), G6 (low pollen) and G7 (high pollen) compared to G4 (CCI_4_) and ↑ levels compared to control G1-3; ↑ liver MDA (nmol/g tissue) in G6 (low pollen) and G7 (high pollen) compared to G5 (silibinin) and control G1-3; ↓ SOD (U/g liver) in G6 (low pollen) and G7 (high pollen) compared to G5 (silibinin) and control G1-3 ↑ AI in G6 (low pollen) compared to G5 (silibinin)and control G1-3 G6 (low pollen) decreased fatty degeneration and regeneration in hepatocytes G7 (high pollen) decreased fatty degeneration	[72]

Note: AA—allyl alcohol; ABTS—2,2’-azino-bis-3-ethylbenzthiazoline-6-sulphonic acid; AI—apoptois index, ALT—alanine aminotransferase; AST—aspartate aminotransferase; ATCC—American Type Culture Collection; BB—bee bread; BP—bee collected pollen; CCI_4_—carbon tetrachloride; CCM—Czech Collection of Microorganisms; COX-1—cyclooxygenase-1, COX-2—cyclooxygenase-2; DPPH—1,1-diphenyl-2-picrylhydrazyl; DW—dry weight,; Eh—96% ethanol extract; El—70% ethanol extract; GAE—gallic acid equivalents, HRSA—hydroxyl radical-scavenging activity; i.p.—intraperitoneal, MDA—malondialdehyde; MIC—minimal inhibition concentration, MBC—minimal bactericidal concentration, Meh—99.9% methanol extract and Mel—70% methanol extract; MNCs—multinucleated cells; MRSA—methicillin resistant *Staphylococcus aureus*, OO—olive oil; PGE_2_—prostaglandin E_2_; PTH—synthetic human parathyroid hormone; SOD—superoxide dismutase; TEAC—Trolox equivalent antioxidant capacity; TFC—total flavonoid content, TPC—Total phenolic content, TRACP—tartrate-resistant acid phosphatase; SO—soy oil, STZ—streptozotocin; VD_3_—1,2-dyhydroxyvitamin D_3_; QE—quercetin equivalents, WSBP—water-solubilized bee pollen extract, ↑—High; ↓—Low.

**Table 3:** In vitro summary of the main studies regarding the effect of BP and BB on multiple cancer and non-cancer cell lines.

BP or BB	Cell Lines	Treatment Schemes	Obtained Results	References
Cernitin T-60 (water-soluble pollen extract with > 90% pollen *w/w*)	Human prostate cell line DU-145	The inhibitory patterns for both the naturally occurring fraction designated as FV-7 in the water soluble component of the pollen extract Cernilton^®^ and an authentic synthetic sample of DIBOA were tested at 1, 10 and 100 g/ml	↓ Growth inhibition (1 μg/mL) of V-7 or DIBOA for day 1–6.; ↑ Inhibitory effect (10 μg/mL) of 50% at day 1 and 80% at day 5; Complete shutdown of the proliferative effects (100 μg/mL) achieved from day 1 to 6	[100]
Chloroform extract from *Brassica rapa* L. BP (CPBC)	Human cancer cell lines (PC-3, lncap, MCF-7, Hela, BEL-7402, BCG-823, KB, A549 and HO8910)	Cell lines treated with various concentrations of CPBC (12.5–100 μg/mL) for 24 h	CPBC remarkably induced concentration-dependent cytotoxicity in PC-3 and lncap cells; 100 μg/mL CPBC could induce cytotoxicity in MCF-7, Hela, BEL7402, BCG-823, KB, A549 and HO8910 cells	[64]
Extracted and fractionated BP polysaccharides from *Rosa × rugosa* Thunb. (WRPP)	Human colon cancer HT-29 and HCT116 cells	Cells treated with varying concentrations (0, 0.5, 2, 5 mg/mL) of various BP polysaccharides for 72 h	Fractions and sub-fractions of WRPP showed a concentration-dependent proliferation-inhibitory effect on HT-29 and HCT116 cells	[29]
BB ethanolic extracts (ebbs) obtained from three different samples of BB from Poland	Glioblastoma cell line (U87MG)	BB extract—effects of EBB1, EBB2, EBB3 (10, 20, 30, 50, 100 µg/mL) on the viability of glioblastoma cell line (U87MG) were studied after 24 h, 48 h and 72 h of treatment.	time-dependent inhibitory effect on the viability of U87MG cells treated EBB; The main inhibitory effect of EBB was observed after 72 h; EBB treatment decreased cell viability to 49–66%.	[81]
*Salix* spp. Beebread (EBB) extract	DASC, U87MG, svgp12	Cytotoxic effect using MTT assay: EBB (50 mg/mL), combination with TMZ (20 mm) on cells after 24 h, 48 h and 72 h of the treatment	↓ Cell viability: EBB = 62.4 ± 4.6% on U87MG after 72 h; ↓ Cell viability: EBB + TMZ: 82.9–85.2% after 48 h and 70.7–80.0% after 72 h on U87MG; ↓ Cell viability: EBB + TMZ: 46.2 ± 3.0% after 72 h on SVGp12	[105]
Date palm pollen (DPP) and volatile esters of fermented and non-fermented *Phoenix dactylifera* L. Pollens (FDPPS)	MCF-7 cell line	Antioxidant activities were determined using DPPH assay, the ferric reducing antioxidant power assay and ABTS assay. Anti-breast-cancer and antiviral activities were determined using the MTT assay	↑ Antioxidant activity of the FDPP extract of 3.16, 3.42, and 2.14 times that of the DPP extract as determined by the ABTS, ferric reducing antioxidant power (FRAP) and DPPH assays; ↑ Anticancer activity of FDPP against the MCF-7 cell line (IC_50_: 9.52 μg/mL) compared with the DPP extract (IC_50_: 96.22 μg/mL); ↑ Antiviral activity of FDPP (CC_50_: 16.5 μg/mL) compared with DPP (CC_50_: 38.8 μg/mL).	[111]
BPE (bee pollen extract)	MCF-7 and L929 cell lines	Antioxidant activities determined with DPPH assay. Antiproliferative activity at different concentrations of BPE and cisplatin was determined using MTT assay on MCF-7 and L929 cell lines.	BPE EC_50_: 0.5 mg/mL; BPE IC_50_: 15 mg/mL on MCF-7 and 26 mg/mL in normal cell line L929; CP IC_50_: 20 μmol/L on MCF-7	[112]
Dimethyl sulfoxide (DMSO) extracts of BP	HL-60 Myeloid Cancer Cell Lines	DMSO extracts of BP were incubated separately with HL-60 cells, and caspase-3 activity evaluated	↑ Apoptosis DMSO extract of pollen (2 mg/mL): 52.2%; ↓ Cell viability: 62%	[106]
Six BB samples (BB1-BB5, BBC)	Human tumor cell lines: MCF-7, NCI-H460, Hela, HepG2; Non-Tumor Porcine Liver Cells: PLP2	In vitro assays—cytotoxicity (ranging from > 400 to 68 µg/mL) on all cell lines	BB1 GI_25_: 164 µg/mL on MCF-7; 345 µg/mL on Hela; BB2 GI_25_: 84 µg/mL on MCF-7; BB3 GI_25_: 164 µg/mL on MCF-7; 253 µg/mL on NCI-H460; 225 µg/mL on Hela; 67 µg/mL on HepG2; BB4 GI_25_: 85 µg/mL on NCI-H460; 209 µg/mL on Hela; BB5 GI_25_: 68 µg/mL on NCI-H460; 276 µg/mL on Hela; BBC GI_25_: 366 µg/mL on Hela; None of the BB samples showed toxicity for normal cells	[104]

Note: A549—lung carcinoma cell lines; BCG-823—gastric adenocarcinoma cells; BEL7402—human liver cancer cell line; CP—cisplatin; DASC—diffuse astrocytoma; DIBOA—hydroxamic acid, 2,4-dihydroxy-1,4-benzoxazin-3-one; DU-145—human prostate cancer cell line; HCT116—human colon cancer cell line; HeLa—human cervix carcinoma cell lines; HepG2—liver hepatocellular carcinoma; HL-60—human leukemia cell line; HO8910—ovarian carcinoma cell lines; HT-29—human colon adenocarcinoma cell line; KB—squamous carcinoma cells; L929—mouse fibroblastic cell line; LNCaP—human prostate adenocarcinoma cells; MCF-7—human breast cancer cell line; MTT—3-(4,5-dimethylthiazol-2-yl)-2,5-diphenyltetrazolium bromide; NCI-H460—human lung cancer cell line; PLP2—Non-Tumor Porcine Liver Cells; SVGp12—normal human astroglia; TMZ—temozolomide; U87MG—human glioblastoma cell lines; ↑—increase; ↓—decrease.

**Table 4:** In vivo studies regarding the effect of BP in multiple diseases which imply liver damage.

Animal Models	The Type of BP, Collection Site and Application Method	Applied Treatment	Effects of BP Administration	References
Eighty male Wistar rats weighing 180–240 g were divided into eight equal groups	Cernitin T60 and Cernitin GBX of specially selected plants, Sweden, orally administered	G1—control; G2—allyl alcohol (AA); G3—AA + Cernitin T60 2.5 mg/kg/day; G4—AA + Cernitin T60 50 mg/kg/day; G5—AA + Cernitin GBX 2.5 mg/kg/day; G6—AA + Cernitin GBX 50 mg/kg/day; G7—AA + Cernitin GBX 2.5 mg/kg/day + Cernitin T60 50 mg/kg/day; G8—AA + Cernitin GBX 50 mg/kg/day + Cernitin T60 50 mg/kg/day.	↑ liver cells apoptosis in G2 ↓ complete cell degeneration in G4 ↓ hepatotoxicity in G1 and G6 ↓ bilirubin level and liver weight; ↓activity ALT and AST in G3 and G4: ↓ serum enzymes activity in G7 and G8	[127]
Forty male mongrel rabbits with initial body weight 3.0–3.8 kg fed with a standard basic diet, randomly divided into four equal groups	Cernitin T60 and Cernitin GBX—from six plant species: Rye grass, Maize, Timothy grass, Pine, Alder flower and Orchard grass; orally administered	G1—control, G2—HFD, G3– HFD + pollen extracts (Cernitin T60—50 mg/kg/24 h + Cernitin GBX—10 mg/kg/24 h) orally, G4—HFD + clofibrate (Pharmaceutical Works ‘Polfa’/25 mg/kg/24 h) orally. HFD = (g/kg/24 h) cholesterol (0.5), hydrogenated coconut oil (l.0), cholic acid (0.1). The experiment lasted 12 weeks	The intima of the aorta of rabbits of G1 (controls) was unchanged. G2, G4 HFD fed groups— ↑ atherosclerotic plaques, the plaque coverage averaging 83.5% compared to only 33.7% in the pollen extract-treated animals. ↑ weight of livers G2 and G4	[128]
S180-bearing mice	*Brassica rapa* L. pollen polysaccharide (LBPP), Wuhan, China; orally administered	G1—normal saline injections. G2—control; G3—polysaccharide LBPP (50 mg/kg body weight); G4—thpolysaccharide LBPP (100 mg/kg body weight); G5—polysaccharide LBPP (200 mg/kg body weight); G6—cyclophosphamid (Cy, 20 mg/kg body weight	↓ growth of S180 (51.26% inhibition rate) with RPP ↓ toxicity RPP + Cy displayed synergism and reduced its toxicity on immune organs. ↑ antitumor activity of RPP ↓ toxicity of RPP on liver, kidney, spleen and thymus	[28]
Eighty male CF1 mice (19–21 g) divided into 8 groups	Bee pollen from mesquite (*Prosopis juliflora* (Sw.) DC.) collected in April in Mexico, extracts of two flavonol concentration (9.794 μg/mL and 21.751 μg/mL), 200 μL orally	G1—provided with cooking oil, G2—200 mL of mesquite BP extract; G3—200 mL of extract of mesquite BP; G4—200 mL of vitamin E (400 UI); G5 intoxicated with bromobenzene—200 mL, 94.211 mg/mL in cooking oil; G6-8 intoxicated with bromobenzene—200 mL, 94.211 mg/mL in cooking oil after the administration of vitamin E (400 UI)	↑ antioxidant activity in vivo on the liver of bromobenzene-intoxicated mice; ↓ MDA in the in vitro biological systems, flavonols (0.07 mg/mL) in mesquite pollen extract; ↓ MDA in the in vivo system, flavonols (21.751 mg/mL); ↓ Liver LPO (the highest dose)	[129]
Female CBA/Hr mice aged 4 months. Experimental and control group consisted of 10 mice each	*Cystus incanus* L. BP from location in Central Croatia’s Dalmatia coast and offshore islands; orally administered to mice	Mice were fed 14 days before testing either with commercial food pellets (control group) or with commercial food pellets mixed with bee pollen (100 mg/kg bw)	↓ TBARS in the liver, but without effect in brain; ↑ AOE in the liver, brain and lysate of erythrocytes; ↓ hepatic LPO; ↑ Apoptosis Hspa9a, Tnfsf6 (liver); ↓ Casp 1 and Cc121c; ↑ SOD, GSH-Px and CAT activity in the lysate of erythrocytes (100 mg/kg bw)	[130]
Male Kunming mice divided into five groups of 12 animals each	*Schisandra chinensis* (Turcz.) Baill. pollen extract (SCPE) from Xi’an, China; administered daily orally for 42 days	In vivo study: SCPE (10, 20 and 40 g/kg) administered to CCl_4_-induced acute liver damage in mice	SCPE—total phenolic content (53.74 ± 1.21 mg GAE/g), total flavonoid content (38.29 ± 0.91 mg Rutin/g); ↓ ALT, AST in acute liver damage induced by CCl_4_, ↓ MDA formation in liver, ↑ SOD and GSH-Px	[131]
Forty-nine 12 weeks old Sprague-Dawley rats divided in seven groups	BP collected during flowering season in Turkey (Western Black Sea region) with dominant component *Castanea sativa* L. (> 45%), 200 mg/kg/day orally, 400 mg/kg/day orally, 7 days	G1—control, 0.9% NaCl (i.p.); G2—control, 0.8 mL/kg olive oil (i.p.); G3—control, 0.8 mL/kg Ethanol (i.p.); G4—CCI_4_, 0.8 mL/kg CCI_4_ in olive oil; G5—Silibinin, 0.8 mL/kg CCI_4_ in olive oil + Silibinin (50 mg/kg/day) gavage; G6—low pollen, 0,8 mL/kg CCI_4_ in olive oil (i.p.) + Pollen (200 mg/kg/day) gavage; G7—high pollen, 0,8 mL/kg CCI4 in olive oil (i.p.) + Pollen (400 mg/kg/day) gavage	↑ AI in G4 compared to G5–G7. Toxicity: ↑ plasma ALT and AST ↑ MDA in liver, RBC and plasma; ↓ SOD in plasma, RBC and liver Pollen administered groups: ↓ Plasma ALT: high dose ↓ Plasma AST ↓ MDA in the plasma, RBC and liver	[72]
Male albino mice divided in: six groups with 11 animals each.	Effects of water extracts of Egyptian bee pollen (WEBP) from Beni-Suef, Upper Egypt, on cisplatin (CDDP) induced hepatic, renal, testicular and genotoxicity in male albino mice; Orally administered	G1—negative control (0.9% NaCl solution by intraperitoneal injection (i.p.) twice/week for 3 weeks). G2—i.p. injection of CDDP (2.8 mg/kg b. wt.) twice/week for 3 weeks. G3—8.4 mg/kg b. wt. of propolis extract oral/day for 14 days. G4—WEBP (140 mg/kg b. wt.) oral/once/day for 14 days. G5 and G6—CDDP injected i.p. with (2.8 mg/kg b. wt. twice/week) alone for 1 week and for the next 2 weeks were given WSDP and WEBP by oral intubation and i.p. injection of CDDP	The treatment of mice with WEBP at a dose of 140 mg/kg b. wt./day, for 14 days with CDDP (2.8 mg/kg b. wt.) resulted in: ↓ Lipid peroxidation in the liver, kidney and testis ↑ CAT and GSH in the liver, kidney and testis The positive control animals taken CDDP alone showed toxic histological and genetical manifestations ↑ LPO in kidney, liver and testis ↓ CAT and GSH in the kidney, liver and testis	[132]
36 adult male Sprague Dawley rats divided into six groups of six animals each	*Schisandra chinensis* (Turcz.) Baill. bee pollen extract (SCBPE) from Jiaozhou, Shandong, China; intragastrically administered (i.g.) in mice	G1—normal saline (10 mL/kg/day) for 12 days and i.p. with saline (10 mL/kg) at the 7th day G2—normal saline (10 mL/kg/day) for 12 days and i.p. CP (8 mg/kg) at the 7th day G3—i.g., VC (400 mg/kg/day) for 12 days days and i.p. CP (8 mg/kg) at the 7th day G4-6—SCBPE (400, 800, 1200 mg/kg/day) for 12 days and i.p. CP (8 mg/kg) at the 7th day	↓ MDA in kidney and dose-dependent in liver ↓ iNOS in liver and dose-dependent in kidney ↑ SOD dose-dependent in liver and kidney ↑ CAT in the liver and kidney ↑ GSH in the liver and kidney	[133]

Note: AI—apoptosis index; ALT—alanine aminotransferase; AOE—modulated antioxidant enzymes; AST—aspartate aminotransferase; CAT—catalase; CCl_4_—Carbon tetrachloride; CDDP—cis-diamminedichloroplatinum(II), cipslatin; Cy—cyclophosphamide; GAE—gallic acid equivalent; GBX—fat-soluble (10–16% of phytosterols) substances; GSH—reduced glutathione; GSH-Px—glutathione peroxidase; HFD—high-fat diet; iNOS—inducible nitric oxide synthase; LPO—lipid peroxides; MDA—malondialdehyde; RBC—red blood cell; SOD—superoxide dismutase; TBARS—thiobarbituric acid reactive substances;; WSDP—water-soluble propolis derivative; ↓—decrease; ↑—increase.

**Table 5:** Enzyme-level changes induced by BB in response to anti-cancer therapies.

Molecules	Organs	Species	Agents	Change	References
**Oxidative stress**					
CAT	Liver, brain, heart	rats	Propoxur	↑	[71]
	kidney	rats	Propoxur	↓	[71]
	Testis	rats	CdCl_2_	↑	[134]
	Plasma	rats	CCl_4_	↓	[135]
	Liver	rats	CCl_4_	↓	[135]
	Liver, kidney, testis	mice	Cisplatin	↑	[132]
	Liver, kidney	rats	Cisplatin	↑	[133]
GST	Liver, kidney, testis	mice	Cisplatin	↑	[132]
GSH	Liver, kidney	rats	Cisplatin	↑	[133]
	Testis, prostate	rats	CdCl_2_	↑	[134]
	Brain	rats	F	↑	[136]
SOD	Liver, kidney, heart, brain	rats	Propoxur	↑	[71]
	Testis	rats	CdCl_2_	↑	[134]
	Liver	mice	CCl_4_	↑	[131]
	Liver	rats	CCl_4_	↑	[72]
	Liver	rats	CCl_4_	↓	[135]
	Plasma	rats	CCl_4_	↑	[135]
	Liver, kidney	rats	Cisplatin	↑	[133]
GSH-Px	Liver, kidney, heart, brain	rats	Propoxur	↑	[71]
	Liver	mice	CCl_4_	↑	[131]
MDA	Liver	mice	Bromobenzene	↓	[129]
	Brain	rats	F	↓	[136]
	Liver, kidney	rats	Cisplatin	↓	[133]
	Liver, kidney, testis	mice	Cisplatin	↓	[132]
	Liver	mice	CCl_4_	↓	[131]
	Liver	rats	CCl_4_	↓	[72]
	Liver	rats	CCl_4_	↓	[135]
	Liver, kidney, heart, brain	rats	Propoxur	↓	[71]
iNOS	Liver, kidney	rats	Cisplatin	↓	[133]
**Inflammation**					
IL-6	Prostate, testis	rats	β-estradiol	↓	[137]
TNF-α	Prostate, testis	rats	β-estradiol	↓	[137]

Note: CAT—catalase; CCl_4_—carbon tetrachloride; CdCl_2_—cadmium chloride; F—fluoride; GSH—reduced glutathione; GSH-Px—glutathione peroxidase; GST—glutathione peroxidase; iNOS—inducible nitric oxide synthase; IL-6—interleukin-6; MDA—malondialdehyde; Propoxur—2-isopropoxyphenyl methylcarbamate; TNF-α—tumor necrosis factor-alpha; ↑—Increased molecular change by chemotherapeutic agents; ↓—Decreased molecular change by chemotherapeutic agents.

**Table 6:** Effects of BP extract treatmens in patients with chronic prostatitis or chronic pelvic pain syndrome.

BP	Patient Disorders	Treatment Schemes	Obtained Results	References
BP extract Cernilton^®^ (several different plants from Sweden)	15 patients with chronic relapsing non-bacterial prostatitis or prostatodynia	Cernilton^®^ administration varied from 1 to 18 months	↑ lasting relief and symptom-free in seven patients ↑ improvement in six patients ↓ response in two patients	[154]
BP extract Cernilton^®^ (several different plants in southern Sweden)	53 patients with benign prostatic hyperplasia (BPH) entered in a double-blind placebo-controlled study	Patients were administered Cernilton^®^ (*n* = 29) and placebo (*n* = 24) in a dose of two capsules for 6 months	↑ subjective improvement with Cernilton^®^ (69%) compared with placebo (30%) ↓ residual urine in Cernilton^®^-treated and in the antero-posterior (A-P) diameter of the prostate on ultrasound	[155]
BP extract Cernilton^®^ R N	treatment of chronic prostatitis syndrome in 90 patients; G1—those without associated complicating factors (CFs) (*n* = 72) G2—those with complicating factors, i.e., urethral structures, prostatic calculi, bladder neck sclerosis (*n* = 18)	Cernilton^®^ R N given in a dose of 1 tablet tid and in most cases treatment was continued for 6 months	↑ response in the group without CFs, 56 (78%); 26 (36%) were cured of their symptoms and signs ↑ in flow rate ↓ in leucocyturia in the post-prostate massage urine (VB3) and ↓ complement C3/coeruloplasmin in the ejaculate in 30 (42%) in the patients with CFs, only one patient showed a response.	[16]
BP extract EA-10, P5 (375 mg/pill)	G1—68 cases of CP G2—63 cases of infertility with CP	G1: group A (*n* = 25): EA-10, P5 + Roxithromycin; group B (*n* = 21): EA-10, P5; group C (*n* = 22): Roxithromycin; G2: group A (*n* = 20): EA-10, P5 + Roxithromycin; group B (*n* = 21): EA-10, P5; group C (*n* = 22): Roxithromycin; Administration twice daily for 4 weeks	Pre-treatment group: ↑ LEPS, MDA, NO ↓ Zn content and SOD; Post-treatment: ↑ LEPS, Zn content and sperm motility and viability ↓ MDA and NO	[156]
Cernilton^®^/Cernitin pollen extract	87 patients: G1: control (*n* = 18); G2: NIH chronic prostatitis category III (*n* = 34); G3: BPH (*n* = 35)	Patients received two capsule daily from 4 to 6 weeks	↑ pain/discomfort domain score (G1: 0.39; G2: 9.79; G3: 1.66) ↑ QoL (G1: 0.39; G2: 8.21; G3: 4.17)	[157]
Prostat/Poltit	115 patients with chronic nonbacterial prostatitis	Each patient was given 1 tablet of prostat (70 mg P5 + 4 mg EA10) twice a day for 8 weeks	↓ NIH-CPSI and QoL ↓ symptom rating scores ↓ WBC counts in prostate massage fluid	[158]
Prostat/Poltit (74 mg highly defined extract of BP from selected *Graminae* spp.)	Two groups: 58 patients between 20 and 55 years old with chronic nonbacterial prostatitis or chronic pelvic pain syndrome were randomized to receive Prostat/Poltit (*n* = 30) or placebo (*n* = 28)	The dose was three tablets/day. The placebo tablets were identical in appearance to the active tablets, but contained no pollen extract.	patients taking Prostat/Poltit: ↑ clinically improvement or symptom-free in 22 patients, compared to ↑ improvement in 10 of the patients taking placebo	[19]
BP extract Cernilton^®^	139 men randomly allocated to the pollen extract (*n* = 70) or placebo (*n* = 69).	Participants were randomised to receive oral capsules of the pollen extract (2 capsules q8 h) or placebo for 12 wk	↑ individual domains pain, QoL and NIH-CPSI score after 12 wk of treatment with pollen extract compared to placebo. Adverse events were minor in all patients studied.	[20]
DEPROX 500^®^ (1 g pollen extract (500 mg per tablet) and vitamins	87 males (25 class IIIa and 62 class IIIb) with a mean age of 33.6 ± 5.9 years with chronic prostatitis/chronic pelvic pain syndrome	Participants were randomised to receive oral capsules of DEPROX 500^®^ (two capsules/day; *n* = 41) or ibuprofen (600 mg, one tablet three times/day; *n* = 46) for four weeks	↓ NIH-CPSI total score by ≥ 25% ↓ Adverse events in the DEPROX 500^®^ treatment group compared to ibuprofen ↑ pain relief and QoL in DEPROX 500^®^ treatment group ↑ antioxidant activity of the pollen extract and protective effect on nerves by vitamins combination	[159]

Note: BPH—Benign Prostatic Hyperplasia; LEPS—leukocytes in expressed prostatic secretion; MDA—malondialdehyde; NIH-CPSI—The National Institutes of Health Chronic Prostatitis Symptom Index; NIH—National Institutes of Health; NO—nitric oxide; QoL—Quality of Well-Being; SOD—superoxide dismutase; WBC—white blood cell count; ↓—decrease; ↑—increase.
